# Therapeutic Potential of Microvesicles in Cell Therapy and Regenerative Medicine of Ocular Diseases With an Especial Focus on Mesenchymal Stem Cells-Derived Microvesicles

**DOI:** 10.3389/fgene.2022.847679

**Published:** 2022-03-29

**Authors:** Lina Moallemi Rad, Alexey V. Yumashev, Bashdar Mahmud Hussen, Hazha Hadayat Jamad, Soudeh Ghafouri-Fard, Mohammad Taheri, Samaneh Rostami, Vahid Niazi, Mohammadreza Hajiesmaeili

**Affiliations:** ^1^ Department of Molecular and Cell Biology, Faculty of Basic Sciences, University of Mazandaran, Babolsar, Iran; ^2^ Department of Prosthetic Dentistry, Sechenov First Moscow State Medical University, Moscow, Russia; ^3^ Department of Pharmacognosy, College of Pharmacy, Hawler Medical University, Kurdistan Region, Erbil, Iraq; ^4^ Center of Research and Strategic Studies, Lebanese French University, Kurdistan Region, Erbil, Iraq; ^5^ Department of Biology, College of Education, Salahaddin University-Erbil, Kurdistan Region, Erbil, Iraq; ^6^ Department of Medical Genetics, School of Medicine, Shahid Beheshti University of Medical Sciences, Tehran, Iran; ^7^ Institute of Human Genetics, Jena University Hospital, Jena, Germany; ^8^ Department of Immunology, School of Medicine, Zanjan University of Medical Sciecnes, Zanjan, Iran; ^9^ Department of Tissue Engineering and Applied Cell Sciences, School of Advanced Technologies in Medicine, Shahid Beheshti University of Medical Sciences, Tehran, Iran; ^10^ Skull Base Research Center, Loghman Hakim Hospital, Shahid Beheshti University of Medical Sciences, Tehran, Iran; ^11^ Critical Care Quality Improvement Research Center, Loghman Hakin Hospital, Shahid Beheshti University of Medical Sciences, Tehran, Iran

**Keywords:** ocular diseases, mesenchymal stem cells, exosomes, regenerative medicine, ncRNAs

## Abstract

These days, mesenchymal stem cells (MSCs), because of immunomodulatory and pro-angiogenic abilities, are known as inevitable factors in regenerative medicine and cell therapy in different diseases such as ocular disorder. Moreover, researchers have indicated that exosome possess an essential potential in the therapeutic application of ocular disease. MSC-derived exosome (MSC-DE) have been identified as efficient as MSCs for treatment of eye injuries due to their small size and rapid diffusion all over the eye. MSC-DEs easily transfer their ingredients such as miRNAs, proteins, and cytokines to the inner layer in the eye and increase the reconstruction of the injured area. Furthermore, MSC-DEs deliver their immunomodulatory cargos in inflamed sites and inhibit immune cell migration, resulting in improvement of autoimmune uveitis. Interestingly, therapeutic effects were shown only in animal models that received MSC-DE. In this review, we summarized the therapeutic potential of MSCs and MSC-DE in cell therapy and regenerative medicine of ocular diseases.

## Introduction

Eye disorders that cause visual impairment include a variety of disorder with severe impacts on health (Flaxman et al., 2017). Treatment of these disorders is complicated in many cases. Recent investigations have shown the beneficial effects of mesenchymal stem cells (MSCs) in treatment of eye disorders. These cells can exert mmunosuppressive, anti-inflammatory, and trophic effects through production of several biological factors (Joe and Gregory-Evans, 2010). These cells produce a wide array of microvesicles, particularly exosomes that carry these biomolecules. The therapeutic effects of MSCs in eye disorders is mainly mediated through secretion of these microvesicle. In this review, we summarized the therapeutic potential of MSCs and these microvesicles in cell therapy and regenerative medicine of ocular diseases.

## Role of Exosomes in Immune-Mediated Ocular Disorder

**FIGURE 1:**
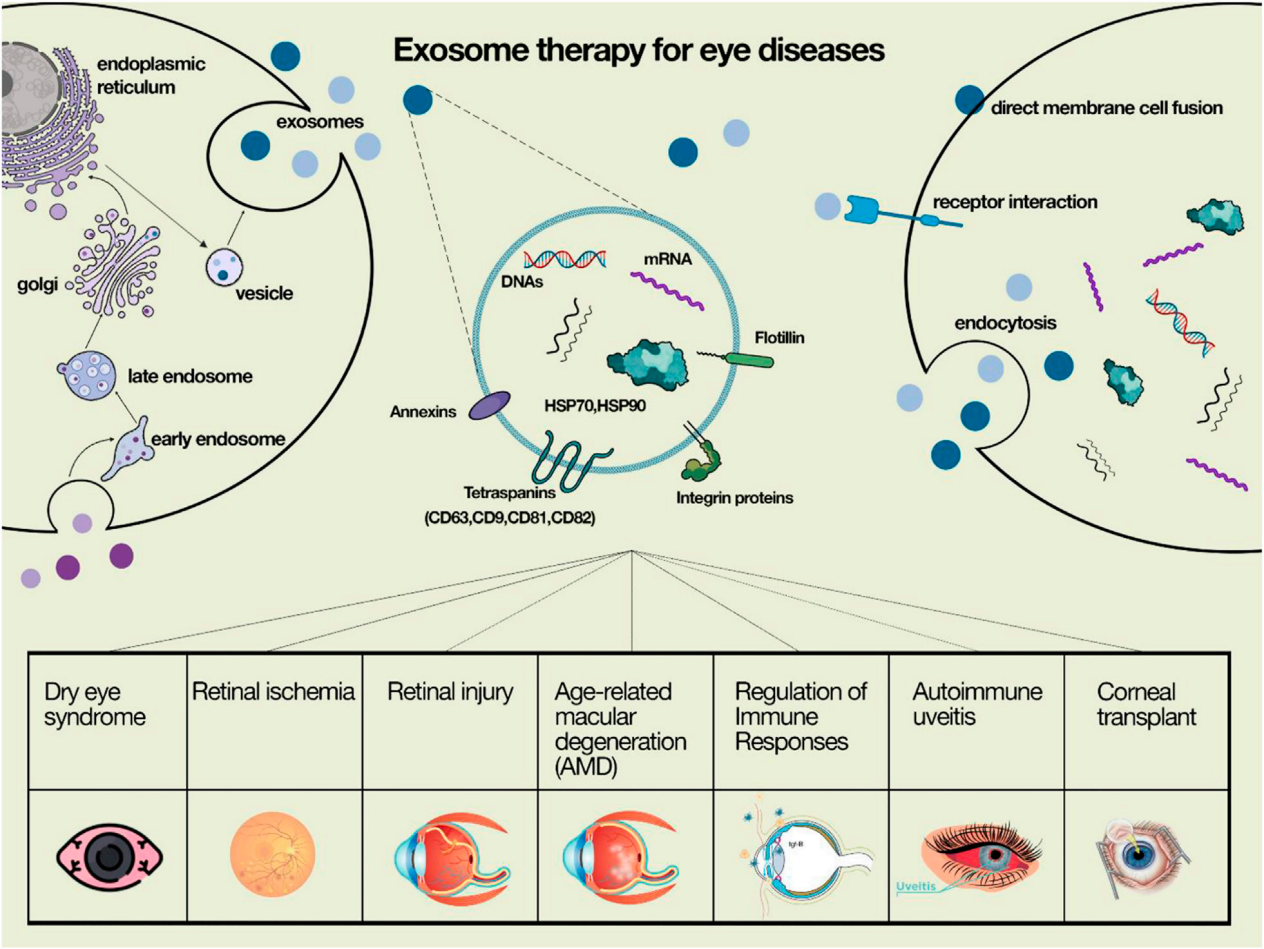
exosomes have been introduced as a novel agent in cell-free therapy in eye diseases.

Exosomes have been introduced as a novel agent in cell-free therapy in eye diseases (Figure 1) In the comong sections, we describe the impact of these agents in each eye disorder.

### Dry Eye or Sjögren’s Syndrome

Sjögren’s syndrome (SS) is an autoimmune disorder which is characterized by infiltration of lymphocyte in salivary glands (SGs) and lacrimal glands (LGs). This disorder is manifested by oral and ocular dryness (Shiboski et al., 2012; López-Miguel et al., 2016). This situation is known as one of the severe dry eye diseases (Bose et al., 2016). Several different pathways such as interferon (IFN) signatures, and NF-kB signalling are responsible for the pathogenesis of SS (Mavragani, 2017; Sandhya et al., 2017). Salivary gland epithelial cells (SGECs) in SS play active roles in the autoimmune and inflammatory responses (Manoussakis and Kapsogeorgou, 2010; Generali et al., 2017). Mainly, lymphocyte T CD4^+^ and B cells invade to epithelial cells (Mavragani et al., 2014; Goules et al., 2017). One study showed that SGECs release some exosomes containing Sm RNPs and Ro/SS-A, which can activate lymphocytes B. This mechanism might signify a mechansim for presentation of intracellular autoantigens to the immune system which might have either an immunogenic or tolerogenic consequence (Kapsogeorgou et al., 2005). In EBV, EBV-miRBART13–3p is transferred from B cells to SGECs by the exosome. EBV-miRBART13–3p targeted aquaporin 5 (AQP5), which can interfere with the secretion of SGs. LG is responsible for the production of tear; thus this disorder is mainly due to immune cells invasion (Li et al., 2019a). One study showed that MSC therapy efficiently alleviated autoimmune dacryoadenitis *in vivo* animal models (Li et al., 2016). MSC-derived exosomes (MSC-DEs) can modulate the immunosuppressive effects and play essential roles in treating eye disorders (Ren, 2019). Recently, it has been shown that injection of MSC-DEs efficiently alleviated inflammation in lacrimal glands compared to those treated with saline. Probably, MSC-DEs provide a promising cell-free therapy for eye disease.

### Corneal Transplant Rejection

The most crucial cause of corneal graft failure is transplant rejection is ascribed to the detection of donor MHC antigens by recipient T cells (Marino et al., 2016; Mahabadi et al., 2018). Exosomes released by donor cells are responsible for rejection and tolerance under certain situations (Gonzalez-Nolasco et al., 2018). It has been revealed that Treg CD4^+^CD25^−^ derived EXs and MSCs-DEs prolong survival of kidney transplants through their specific components (Wen et al., 2016; Aiello et al., 2017). These data suggest that exosomes can serve as a cell-free therapy to increase immune tolerance in corneal transplantation. Due to the absence of enough donated corneas, other alternatives such as collagen gels, synthetic polymers, tissue-engineered scaffolds has also been introduced (Gain et al., 2016; Williams et al., 2018). Epithelial cell-derived exosomes increase the proliferation of fibroblast and alleviate wound healing in corneal injuries (Han et al., 2017). It has been shown that limbal keratocytes-derived exosomes activate Akt signalling and increase wound healing in limbal epithelial cells (Leszczynska et al., 2018; Ghafouri-Fard et al., 2021a). Another study has revealed that corneal mesenchymal stem cell-derived exosomes could increase wound healing in corneal injuries (Samaeekia et al., 2018). Recently, it has been shown that exosomes have an essential role in corneal injury and regenerative medicine of eye disease (Basu and Ludlow, 2016).

### Autoimmune Uveitis

Autoimmune uveitis is described as inflammation of the uvea tissue and adjacent tissues (Papotto et al., 2014). This autoimmune disease is mostly due to the unsuitable function of T-cells (Krishna et al., 2017). Th17 lymphocytes and other innate immune cells such as natural killer (NK) cells and dendritic cells (DCs) mediate autoimmune uveitis by secretion of various cytokines (Caspi, 2010; Chong et al., 2015; Patel and Kuchroo, 2015; Pepple and Lin, 2018). Retinal pigment epithelium (RPE) cells may be destroyed in inflammatory actions in posterior uveitis (Sevgi et al., 2017). RPE cells could release exosomes as an immunosuppressive agent and suppress Th17 and Th22 cells (Shi et al., 2015). According to the immunoregulatory effect of RPE-derived exosomes, they may be used as a therapeutic agent to treat uveitis in the future. It has been shown that bone marrow MSCs-DEs could alleviate autoimmune uveoretinitis (Shigemoto-Kuroda et al., 2017). It has been proven that cord blood MSCs derived EXs (CB-MSC-DEs) can not inhibit the function of T cell lymphocytes, but they suppress the migration of immune cells (Bai et al., 2017a). The mentioned result suggested that MSC-DEs have therapeutic application in the treatment of autoimmune uveitis. Further investigation is necessary to reveal the anti-inflammatory and immunomodulatory mechanisms of these processes.

### Age-Related Macular Degeneration

One of the reasons for blindness in older adults is AMD, a multifactorial disease (Wong et al., 2014). AMD is divided into 1) early non-exudative and 2) late exudative forms (Pennington and DeAngelis, 2016). It has been proven that the complement system and inflammatory elements have an essential role in the pathogenesis of AMD (Bora et al., 2015; Perez and Caspi, 2015). Point mutations in complement factor H, the regulator of the complement pathway, or deficiency of protein C3 complement enhance the risk of developing AMD (Liszewski et al., 2017; Toomey et al., 2018; Park et al., 2019). It has been shown that RPE cell-derived exosomes are responsible for the formation of innate immunity in AMD. In exudative AMD, especially in the choroidal neovascular membranes, macrophages are the main cells that attract inflammatory cells (Nussenblatt et al., 2013). Vascular endothelial growth factor (VEGF) and macrophages polarization have been determined as essential mechanisms that trigger pathologic neovascularization in AMD (Sene et al., 2015). It has been proven that MSC-DEs could regulate macrophage polarization, decrease secretion of VEGF, control abnormal neovascularization and, alleviate pathogenesis of AMD (Pakravan et al., 2017; Li et al., 2019b).

Table 1 summarizes the role of exosome in eye disease.

**TABLE 1:** Role of exosome in eye disease.

disease	Origin of exosomes	Ingrediants	Mechanisem	References
SS	Salivary gland epithelial cells	Autoantigenic Ro/SS-A, La/SS-B and Sm RNPs	Induce immune response	Kapsogeorgou et al. (2005)
SS	EVB-infected B lymphocytes	miR-BART13–3p	Impair salivary gland function	Gallo et al. (2016)
Laser injury	UCB-MSCs	*	Healing of laser injuries	Yu et al. (2016)
AMD	ARPE-19 cells	Cytokeratin 8, Hsp70, myosin-9	Diagnose neovascular AMD	Kang et al. (2014)
AMD	RACs	Antiangiogenic factors	Suppresses LICN laser-induced choroidal neovascularization	Hajrasouliha et al. (2013)
Diabetic retinopathy	AD-MSCs	*	Decrease retina degeneration	Safwat et al. (2018)
Retinal ischemia	UCB-MSCs	*	Decrease apoptosis of retinal cells	Mathew et al. (2019)
AMD	RPE	*	Used as a drug delivery vesicles	Aboul Naga et al. (2015)
AMD	Serum	miR-486–5p, miR-626	Diagnose AMD as biomarker	Elbay et al. (2019)
AMD	ARPE-19 cells under oxidative stress	Signaling phosphoproteins	Mediate cell-cell signaling	Biasutto et al. (2013)
AMD	ARPE-19	Complement protein C3	Interact with the complement pathways	Wang et al. (2009)
AMD	ARPE-19 cells	VEGFR2	Increase angiogenesis	Atienzar‐Aroca et al. (2018)
DR	Retinal photoreceptors	miRNAs, VEGF	Modulate angiogenesis	Maisto et al. (2020)
Corneal damaged	AD-MSCs	*	Enhance proliferation of corneal stromal cells	Shen et al. (2018a)
Glaucoma	BM-MSCs	*	Increase neuroprotection	Mead et al. (2018)
DR	PRP	CXCL10	Upregulates the TLR4 signaling pathway	Zhang et al. (2019a)
DR	Pancreatic-β-cells	miR-15a	Enhance diabetic complications	Kamalden et al. (2017)
EAU	UCB-MSCs	*	Inhibit migration of inflammatory cells	Bai et al. (2017b)
DR	UCB-MSCs	miR-126	Suppress HMGB1 signaling pathway	Zhang et al. (2019b)
DR	Diabetic mice’s plasma	Ig-G	Activation of complement pathway	Huang et al. (2018)
DR	MSC under hypoxic conditions	*	Decrease severity of retinal ischemia	Moisseiev et al. (2017)
DR	Plasma	PPARγ	Play essencial role in proliferative DR	Katome et al. (2015)
Optic nerve injury	BM-MSCs	*	Increase RGC survival	Mead et al. (2016)
Corneal implant	pig corneal epithelium	*	Promote corneal regeneration	angamreddy et al. (2018)
	cells			
Corneal wound healing	corneal epithelial cells	Thrombospondin-2 and C-X-C motif chemokine 5	Angiogenesis, increase keratocyte proliferation	Han et al. (2017)
Corneal wound healing	human cornea limbal	Small RNAs	Increase proliferation and wound healing	Leszczynska et al. (2018)
	keratocytes			
Corneal wound healing	Human corneal MSCs	*	Increase corneal Epithelial wound healing	Samaeekia et al. (2018)
Autoimmune uveoretinitis	BM-MSCs	*	Suppressing Th1/Th17 and Tcell development	Shigemoto-Kuroda et al. (2017)
Autoimmune uveoretinitis	UCB-MSCs	*	Inhibiting inflammatory cell migration	Bai et al. (2017a)
Hyperglycemiainduced retinal inflammation	UCB-MSCs	miR-126	Downregulating HMGB1 signaling	Zhang et al. (2019b)

UCB-MSCs, Umbilical cord blood mesenchymal stem cell; SS, Sjögren’s syndrome; EVB, Epstein-Barr virus; BM-MSC, Bone marrow mesenchymal stem cell; AMD, age-related macular degeneration; HMGB1, high-mobility group box 1; ARPE-19, human retinal pigment epithelium cell line; DR, Diabetic retinopathy; PPARγ, peroxisome proliferator-activated receptor gamma; AMD, Age-related macular degeneration; RACs, Retinal astroglial cells; RPE, Retinal pigment epithelial; LICN, laser-induced choroidal neovascularization; RGC, retinal ganglion cell; EAU, autoimmune uveitis; AD-MSCs, adipode-derived mesenchymal stem cell.

### Regulation of Immune Responses in Eye Diseases

MSCs release a wide range of extracellular vesicles, e.g., microvesicles and exosomes (Yeo et al., 2013). MSC-DEs play an essential role in physiological and pathological processes such as cell-cell communication, modulation of inflammation, and immune response (Théry et al., 2009; Zöller, 2009). Amniotic fluid MSC-DEs are enriched by TGF-β as one of the most immunosuppressive factors. TGF-β inhibits cell cycle of T cells and decreases adaptive immunity and T cell-mediated inflammation (Di Nicola et al., 2002; Volarevic et al., 2017). Also, TGF-β could suppress the function of B cells and decrease the production of antibodies (Balbi et al., 2017). Interestingly, amniotic fluid MSC-DEs can not inhibit the function of CD4 + CD25 + FoxP3+ T regulatory cells, proving the importance of immunosuppressive and therapeutic application of amniotic fluid MSC-DEs in inflammation. Recently, an ophthalmic solution has been introduced that contains IL-1 receptor antagonist (IL-1Ra) and amniotic fluid MSC-DEs. Due to its immunomodulatory effect, this product has a therapeutic application in treating dry eye syndrome (DES) and corneal injuries. Corneal injuries are generally induced by inflammation and engagement of immune cells (Dana et al., 2000). IL-1Ra attaches to IL-1 receptor and inhibits activation of IL-1R and subsequently prevents secretion of various pro-inflammatory cytokines, chemokines, and invasion of immune cells in injured corneas (Balbi et al., 2017). In DES inflammation, DCs and Th17 cells participate in the pathogenesis of the disease. (De Paiva et al., 2009; Théry et al., 2009). Amniotic fluid MSC-DEs cargo immunomodulatory factor, increase the number of regulatory DCs, decrease maturation of DC and attenuate the immune response (Gayton, 2009; Yi and Song, 2012; Nakamura et al., 2015; Merino-González et al., 2016).

## Exosome Therapy in Retinal Injury

It has been indicated that ischemia, infection and other injuries can damage retinal cells and result in visual dysfunction (Yoles and Schwartz, 1998). Recently, it has been shown that exosome and MSCs have a therapeutic role in treating retinal disorders. MSC-DE and MSCs therapy can modulate the immune response and cytokine levels in laser-induced retinal injury (LIRI). Also, MSC-DEs decrease inflammation and CD68 ^+^ macrophages and diminish apoptosis of retinal cells (Yu et al., 2016). MSC-DEs modulate secretion of different chemokines such as TNF-α and MCP-1 in retinal injuries and decrease monocytes migration (Yu et al., 2016). Another study showed that bone marrow MSCs derived exosomes (BM-MSC-DE) have therapeutic potential in the regeneration of retinal ganglion cells (RGCs) in optic nerve crush (Mead and Tomarev, 2017). BM-MSC-DE increases survival and preserves the function of RGCs in animal models. Furthermore, due to the diffusion of BM-MSC-DE into the inner retina, they have shown more therapeutic effects than BM-MSCs therapy that remain in the vitreous after injection (Yu et al., 2016; Mead and Tomarev, 2017).

MSCDEs increase neuroprotection of retinal cells after heat-induced injuries in animal models (59). MSC-DEs also decrease MCP-1 in retinal cells, consequently suppressing migration of macrophages and microglial cells toward the injured area and diminish degeneration. *In vitro*, retinal pigment epithelium (RPE) injured by laser produces a large amount of VEGF. Conversely, UC-MSC-DE down-regulates VEGF expression in laser damage RPE cells (Huang et al., 2018). In addition, it has been demonstrated that retinal astrocyte-derived exosomes can suppress neovascularization (Hajrasouliha et al., 2013).

Retinal ischemia occurs by an obstruction in blood vessels or detachment of the retina resulting in severe damage such as blindness. It has been indicated that MSCs therapy can inhibit degeneration of the retina in this process (Dreixler et al., 2014). Moisseiev et al. have demonstrated that BM-MSC-DE could simulate the therapeutic effect of MSCs in the treatment of retinal ischemia (Moisseiev et al., 2017). Furthermore, it has been shown that exosomes therapy inhibit microglia/astrocyte activation and subsequently enhance the secretion of pro-inflammatory cytokines such as IL-1β and IL-6 and afterwards decrease retinal ganglion cells (van der Merwe et al., 2019). Also, it has been indicated that intravitreal injection of exosomes could decrease dysfunction of retinal ganglion cells (van der Merwe et al., 2019). In the animal model, BM-MSC-DEs decrease secretion of pro-inflammatory cytokines and, increase autophagy (Ma et al., 2020). Although the exact mechanism of exosome therapy in the regeneration of the retina is not completely clear, the role of the exosome component on the neuroprotective and anti-inflammatory process has been proved.

## Exosome Therapy in Corneal Injuries

Mechanical/chemical damage, immune responses and genetic disorders can induce corneal injuries with inflammation and neovascularization. Therefore, delayed or inappropriate medical intervention may lead to blindness. Recently, MSC therapy has been demonstrated to possess an influential role in reducing inflammation and modulating anti-angiogenesis in corneal injuries. Likewise, the anti-inflammation, anti-angiogenesis, and wound repair potential of MSC-DEs in different tissue models have been illustrated. Thus, these therapeutic strategies may effectively promote corneal wound repair by affecting inflammation, angiogenesis, and tissue regeneration. Furthermore, during the culture of corneal stromal cells with adipose MSC-DEs, corneal stromal cells manifested increased proliferation and inhibited apoptosis with grated deposition of collagen (Shen et al., 2018b).

Furthermore, in the stromal wound caused in an animal model, corneal stromal stem cell-derived exosomes alleviated inflammation by suppressing neutrophil infiltration (Hertsenberg et al., 2017). Also, a corneal epithelial wound was treated by corneal stromal stem cell-derived exosome (Samaeekia et al., 2018). Thus, mentioned studies propose the importance of MSC-DE in ocular and corneal disorders.

### MSCs Therapy in Corneal Regeneration

**FIGURE 2:**
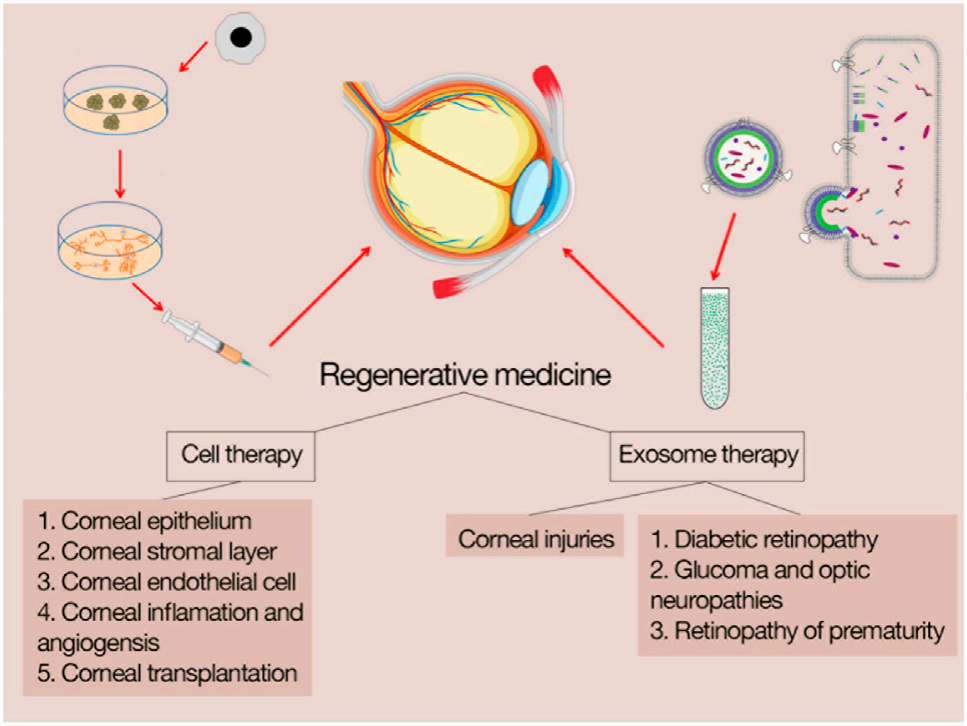
cell therapy and exosome therapy in corneal disease.

MSCtherapy in the regeneration of corneal tissue is related to two mechanisms; 1) direct cell replacement (Karp and Leng Teo, 2009) and 2) paracrine effect of regulating immune response, modulating inflammation and adjusting wound repair (Phinney and Prockop, 2007; Burrello et al., 2016). Figure 2 shows the outlines of cell therapy and exosome therapy in corneal diseases.

### MSCs Therapy in Cornea Epithelium

CE includes several layers of stratified squamous that are located in the most outer part of the cornea. Physical trauma, infection, and limbal stem cell deficiency can break the structure of CE and cause continuous defects, such as neovascularization, opacities, and immune response that result in corneal blindness (Dua et al., 2005). It has been demonstrated that MSCs can transdifferentiate into epithelial cells and other neuroectoderm-derived cells, for example, astrocytes and neurons (Phinney and Prockop, 2007). Mesenchymal-epithelial-transition (MET) and epithelial-mesenchymal transition (EMT) have a critical role in organogenesis and regenerative medicine in animal models (Kim et al., 2017; Shao et al., 2018; Sivagurunathan et al., 2018). Coculture of BM-MSC with limbal stem cells resulted in polygonal epithelial-like cells with overexpression of CK3 and CK12 (corneal epithelium-specific cytokeratin 3) (Gu et al., 2009; Jiang et al., 2010).

On the other hand, AD-MSCs cultured in the supernatant of CE cell culture indicated epithelial-like cells with overexpression of CK3 and CK12 (Nieto-Miguel et al., 2013). Nevertheless, transplantation of BM- MSCs on injured corneas did not affect expression of corneal epithelium-specific cytokeratin (Ma et al., 2006). Conversely, the combination of BM-MSCs with fibrin gel to injured corneal shows an excellent therapeutic effect and manifested upregulation of CK3 (Gu et al., 2009). Recently, the application of small molecules has revealed much concentration to manipulate target gene reprogramming and change the cellular fate in the MET process (Teng et al., 2015; Ghosh et al., 2018). Epithelial progenitors derived from adipose MSC induce overexpression of N-cadherin, E-cadherin, and feature of MET progression. The mixture of these progenitors with fibrin gel can improve transparency and increase the healing of corneal injuries. MSCs can be accessible for treating corneal epithelial injuries and regenerative medicine in ocular disorders. Also, transplantation of limbal epithelial cells combined with amniotic membrane or fibrin gel is another procedure for severe limbal stem cell deficiency (Basu et al., 2012; Rama et al., 2017).

Furthermore, it has been shown that *ex vivo* expansion of limbal derived epithelial stem cells possesses a theoretical advantage for treating an ocular surface disorder (Teng et al., 2015; Ghosh et al., 2018). The efficacy of MSC therapy has been compared with cultivated limbal epithelial cells and the result indicated both could reconstruct corneal epithelial disease (Calonge et al., 2019). The mentioned result has manifested the application of MSC in the regeneration of corneal epithelial disorder. However, further evaluation is necessary to explain other aspects of the therapeutic procedure.

### MSCs Therapy in Corneal Stromal Layer

CSL, the thickest corneal layer, consists of different components such as collagen fibrils, specialized extracellular matrix (ECM), etc. Corneal stromal keratocytes (CSKs) along with keratan sulfate proteoglycans, stromal crystallins, collagenous lamellae and ECM proteins have a critical role in the transparency and integrity of CSL (West-Mays and Dwivedi, 2006). Usually, mechanical damage and disease induce the death of CSK, resulting in decreased proteoglycan synthesis and disruption of collagen fibrils. In these situations, surviving CSKs around the damaged site are activated and start treating stromal wound healing. Unfortunately, some CSKs transform into highly contractile myofibroblasts and form corneal opacities and scars. These opacities can interfere with the transmission of light, resulting in blindness. Several studies have demonstrated that the differentiation of BM-MSCs and UCB-MSCs into keratocyte-like cells can reconstruct corneal stromal clarity (Liu et al., 2010; Liu et al., 2012; Park et al., 2012). Lumican knockout (Lum−/−) animal model with deformation of collagen fibrils show stromal opacities (Kao and Liu, 2002). However, injection of UCB-MSCs can recover disturbed collagen and restore corneal transparency with the formation of CSK-like cells and inhibition of the inflammatory reaction (Liu et al., 2010). Corneal stromal-derived stem cells and MSCs showed similar features and have been shown to have the same potential for regeneration of stromal tissue, decrease scarring and inhibition of inflammation (Du et al., 2009; Kureshi et al., 2014; Funderburgh et al., 2016; Hertsenberg et al., 2017). It has been shown that dental MSCs possess an essential role in regenerative medicine. Among the identified dental stem cells, periodontal ligament and dental pulp derived stem cells due to similar origin with CSKs and neural crest, have the same developmental pathway (Gronthos et al., 2000; Seo et al., 2004; Huang et al., 2009; Yam et al., 2015). Therefore, MSC therapy could be a beneficial procedure for the treatment of severe keratoconus eyes. However, further study will be necessary to explain the functionality of MSCs and delineate their translational potential for therapeutic application in treating stromal diseases.

### MSCs Therapy in Corneal Endothelial Cells Regeneration

The CEC is the most inner layer of the corneal that consists of a hexagonal cells monolayer (Bourne, 2003). Mature CECs have an active ATPase pump that transport ions from CSL to aqueous humour and regulate hydration level and oedema in CSL, resulting in normal vision (Bonanno, 2012). Due to the overexpression of negative cell cycle regulators and mitogenic inhibitors, CECs have been introduced as non-mitotic cells (Joyce, 2012). Therefore, mechanical damage, ageing, and disease can lead to stromal oedema, CEC dysfunction, and disturbance in normal vision. Recently, cell therapy and corneal transplantation have been introduced as alternative therapies (Kinoshita et al., 2018). MSCs can be used as beneficial therapeutic tools for CEC production *in vitro* and treating CEC diseases such as Fuchs’ endothelial dystrophy (Joyce et al., 2012). Recent studies have shown differentiation and generation of endothelial-like cells through UC-MSC (Yamashita et al., 2018). Further studies are necessary to indicate the application of MSC in CSL regeneration.

### MSCs Therapy in Corneal Inflammation and Angiogenesis

It has been shown that MSCs have anti-inflammatory potential and angiogenic ability which are important in treatment of corneal diseases. BM-MSCs could alleviate injuries and regulate angiogenesis in the animal models (Ma et al., 2006; Ye et al., 2006; Jiang et al., 2010). MSCs therapy in corneal disease inhibits infiltration of immune cells such as CD68^+^ macrophages and decreases pro-inflammatory cytokines, such as IL-2, IL-17, VEGF and matrix metallopeptidase 2 (MMP2). Cell therapy in corneal enhances anti-inflammatory cytokines, for example, IL-6, IL-10, thrombospondin-1 (Ma et al., 2006; Ye et al., 2006; Jiang et al., 2010).

Mentioned anti-inflammatory and pro-inflammatory cytokines modified the corneal environment, increasing wound healing in injured sites (Yao et al., 2012). In addition, MSCs exert an angiogenesis effect by secretion of innumerable pro-angiogenic and anti-angiogenic factors. However, MSCs increase the expression of TSP-1 that enhances endothelial cell apoptosis, suppresses angiogenesis and decreases MMP2 (Oh et al., 2008; Kaur et al., 2010; Bazzazi et al., 2018; Ahani-Nahayati et al., 2021a). Thus, it has been demonstrated that corneal stromal stem cells (CSSCs) have the effective potential for the regulation of inflammation and wound healing (Pinnamaneni and Funderburgh, 2012).

### Importance of MSCs Therapy in Corneal Transplantation

Several studies have demonstrated the roles of MSC in the survival of corneal after transplantation (Oh et al., 2008; Casiraghi et al., 2012; Oh et al., 2012). MSCs have different immunomodulatory features that inhibit the expansion and activation of antigen-presenting cells (APCs), NK cells and other cytotoxic cells (De Miguel et al., 2012). Likewise, MSCs trigger the secretion of IL-10 from immature APCs and increase T-cell inhibitors that modulate the immune response (Beyth et al., 2005; Oh et al., 2012). Decreased mature APC population increase immune tolerance during MSCs therapy (Casiraghi et al., 2012; Oh et al., 2012). It has been illustrated that MSCs therapy suppresses expansion, cytokine secretion, and maturation of T/B-cells and modulates T regulatory cells generation (Crop et al., 2009; Casiraghi et al., 2014; Coulson-Thomas et al., 2014; Ghafouri-Fard et al., 2020). Finally, MSCs have a membrane covered by anti-inflammatory molecules, glycocalyx and versican, which regulate the host inflammatory immune response (Coulson-Thomas et al., 2014; Kao and Coulson-Thomas, 2016).

## Exosome Therapy in Other Ocular Disorders

### Diabetic Retinopathy

Diabetic retinopathy (DR) is one of the most critical complications of diabetes caused by high glucous levels, resulting in microangiopathy and injuries in the back of the eye (retina). DR is classified into three pathological stages; 1) non-proliferative, 2) pre-proliferative, 3) proliferative. Vascular leakage in diabetic persons induces macular oedema, a subtype of non-proliferative of DR (Stitt et al., 2016; Wong et al., 2016). The pre-proliferative stage is associated with the occlusion of blood vessels and the manifestation of the non-perfusion region. Neovascularization is one of the most important criteria in proliferative DR (Stitt et al., 2016; Wong et al., 2016). It has been demonstrated that hypoxia plays an essential role in the proliferative DR stage (Wong et al., 2016). Retinal ischemia-reperfusion injury (IRI) is associated with the complications of DR (Stitt et al., 2016; Wong et al., 2016). IRI increases the production of reactive oxygen species (ROS) that causes necrosis, apoptosis resulting in neurons and RGCs death (Liu et al., 2016; Yamashita et al., 2018). MSC-DE could improve complications of DR (Zhang et al., 2019b). Exosomes containing angiogenic active cargo have been indicated to treat diabetic microangiopathy and retinal ischemia in animal models (Moisseiev et al., 2017). A study has shown that a high level of glucose induces DR phenotype in retinal photoreceptors. In addition, a high level of glucose enhances VEGF and decreases anti-angiogenic miRNA in photoreceptors and exosomes (Maisto et al., 2020). Recently, researchers have shown that AD-MSC containing miR-192 or miR-222 has therapeutic application in treating DR and can inhibit inflammatory response and angiogenesis (Safwat et al., 2018). Likewise, injection of UC-MSC-DE with overexpression of miR-126 in a diabetic animal model has been shown to suppress HMGB1 signalling pathway and decrease inflammation caused by diabetes (60).

### Exosome Therapy in Glaucoma and Optic Neuropathies

Glaucoma is an ocular disorder characterized by degeneration of the optic nerve, resulting in a visual disturbance. Glaucoma is usually because of insufficient blood supply and/or increased intraocular pressure (IOP). Glaucoma is classified into two groups 1) open-angle glaucoma (primary) 2) closed-angle glaucoma (secondary) (Davis et al., 2016). Patients with open-angle glaucoma possess normal or elevated IOP without and identified reasons (Razeghinejad et al., 2011; Weinreb et al., 2014; Davis et al., 2016).

Conversely, secondary glaucoma has several reasons, such as increased IOP (Razeghinejad et al., 2011; Weinreb et al., 2014; Davis et al., 2016). In glaucoma, increased IOP damages RGCs resulting in a visual interruption (Almasieh et al., 2012). Therefore, the essential method in treating glaucoma is decreasing intraocular pressure that diminishes the impairment of RGCs. Drain aqueous humour by trabecular meshwork (TM) has been defined as clinical treatment when intraocular pressure increases (Harrell et al., 2019). It has been demonstrated that treating TM by pigmented ciliary epithelium (NPCE) derived exosomes down-regulates Wnt signalling and expression of COL3A1.

Furthermore, these studies showed that mentioned exosomes could modulate the visual water system (Yang et al., 2020). Moreover, MSC-Des have therapeutic application in glaucoma and traumatic optic neuropathies. MSC-DEs have been shown to increase the survival of RGCs, regeneration of axons and exert neuroprotective effect in an animal model (Mead and Tomarev, 2017; Mead et al., 2018).

### Exosome Therapy in Retinopathy of Prematurity

ROP is one of the reasons that cause blindness in premature infants. ROP is characterized by retinal vasculature abnormalities that involve the retina (Fulton et al., 2009; Kong et al., 2012; Hansen et al., 2017). This disease is divided into two oxygen-dependent phases; 1) inhibition of vascular growth 2) vascular proliferation (Hellström et al., 2013). In phase 1, hyperoxia (increased oxygen pressure) suppresses the secretion of VEGF and is the main inducer of disease (Hellström et al., 2013). In phase 2, the baby has grown, and metabolism increases, resulting in a hypoxic state and enhancing VEGF expression (Hellström et al., 2013). It has been shown that MSC-DEs can modulate hyperoxia-induced retinopathy, suggesting a new safe non-cellular therapy for ROP (Moisseiev et al., 2017). Also, researchers have demonstrated the influential role of microglia derived-exosome in treating retina-related diseases (Ebneter et al., 2017; Xu et al., 2019; Ahani-Nahayati et al., 2021b). Injection of microglia derived exosome into vitreous has induced down-regulation of VEGF, TGF-β and suppresses apoptosis of photoreceptor, indicating the therapeutic application of exosome in ROP (Xu et al., 2019).

### Uveal Melanoma

UM is intraocular cancer that involves the choroid, ciliary body, or iris (Chattopadhyay et al., 2016; Lande et al., 2020). Nearly more than fifty percent of patients with UM possess organ metastases. Metastasis in UM is a common phenomenon and usually involve the liver (Carvajal et al., 2017). Isolation of exosomes from local blood circulation in patients with liver involvement indicated UM origin of exosomes with enrichment of Melan-A and melanoma-related microRNAs (Eldh et al., 2014). It has been manifested that isolated exosomes from patients with UM are a specific diagnostic marker (Ragusa et al., 2015). However, exosomes have not been thoroughly examined in UM; for this reason, more studies are necessary to show their importance in eye malignancies.

### Importance of Exosomes in Angiogenesis

In the last decade, it has been shown that the exosome has an essential role in angiogenesis. For example, several studies demonstrated that the injection of hematopoietic stem cells CD34 ^+^ stem cell-derived exosomes (HSC-DE) into the limb ischemia animal model increases the level of miRNA-126–3p and angiogenesis (Mathiyalagan et al., 2017; Mathiyalagan and Sahoo, 2017; Sahoo et al., 2017; Ghafouri-Fard et al., 2021b). Furthermore, pericardial effusion-derived exosomes can enhance angiogenesis in patients with heart failure (Vicencio et al., 2015; Beltrami et al., 2017). Moreover, it has been manifested that MSC-derived exosomes can decrease VEGF and inhibit angiogenesis (Lee et al., 2013). Retinal ischemia is the main trigger of the proliferative stage in DR, which increase neovascularization. Drug prescription and surgery can not alleviate this issue. However, today there is a therapeutic approach that exosome has treating potential in neovascularization (Stępień et al., 2012).

## Conclusion and Future Perspectives

Although cell therapy with MSCs have a critical role in treating disease, recent studies demonstrated that cell-free therapy with MSC-DE has more advantages than MSCs. MSCs-therapy has several side effects such as allogeneic reaction, unexpected differentiation and impede of microvascularization, whereas exosome therapy is safe for these problems. Exosome therapy has crucial therapeutic applications as MSCs without mentioned side effects. Moreover, exosomes can transfer their component through biological barriers and deliver their cargo to target organs because of their small size (Yu et al., 2016; Niazi et al., 2021).

Nevertheless, further evaluations are necessary to solve some present challenges. For example, freezing and thawing, investigating the effects of lyophilization, optimum dose and injection intervals to keep the long-lasting effects of exosome in eye disease are essential issues that need more examination. In addition, the possibility of toxicity in higher dosage levels injection should be determined. Moreover, exosomes are extremely heterogeneous depending on the source of MSC and the origin of MSC is associated with the therapeutic effect of exosomes in eye disease.
